# Corticosteroid therapy in Duchenne muscular dystrophy: Management and new insights

**DOI:** 10.1111/dmcn.16485

**Published:** 2025-09-04

**Authors:** Claudia Brogna, Eugenio Mercuri

**Affiliations:** ^1^ Pediatric Neurology Unit Università Cattolica del Sacro Cuore Rome Italy; ^2^ Pediatric Neurology Unit, Fondazione Policlinico Universitario “A. Gemelli” IRCCS Rome Italy

## Abstract

**This commentary is on the original article by Schiava et al. on pages** 429–440 **of this issue.**

Duchenne muscular dystrophy (DMD) is an X‐linked neuromuscular disorder caused by the absence of dystrophin protein, leading to progressively significant muscle degeneration with loss of ambulation and progressive respiratory and cardiac insufficiency, affecting quality of life and survival.^1^ In the last few decades, several therapeutic approaches have targeted dystrophin restoration using gene therapy or exon skipping; while others have focused on the peripheral mechanisms of fibrosis and inflammation. Some of these approaches have been approved in different countries and although none of them appears to be able to halt or reverse the progression of the disease, there are encouraging results suggesting that they may reduce disease progression.

The advent of new synthetic dissociative steroids (such as vamorolone) and the use of a double dose of glucocorticoid at the time of treatment with gene therapy have revived the interest in glucocorticoids. Although glucocorticoid therapy is recommended as standard of care for DMD, there is still no consensus on the regimen and the dosage to be used. It is only recently that the different regimes have been systematically explored in a large international effort.^2^ This study by Guglieri et al. also provided additional information on different aspects related to function and possible adverse events.^2^

Schiava et al.^3^ explored the relation between anthropometric measures (height, weight, body mass index [BMI] z‐scores) and commonly used motor assessments (rise from supine velocity, 10‐m walk/run velocity, North Star Ambulatory Assessment, 6‐minute walk test) in a cohort of 194 young males with DMD. The cohort was aged between 4 to 7 years and treated with different regimes of glucocorticoids during a follow‐up of 3 years and 5 years. The authors found that height z‐score at glucocorticoid initiation, rather than age of initiation, was a significant predictor of height trajectory and that the type of glucocorticoid regimen influenced height and weight trajectories but not BMI. They also found that older age of glucocorticoid initiation was associated with increased weight gain (*p* = 0.001). Changes in height and weight z‐scores significantly correlated with clinical outcomes.

These findings are important and provide a structured approach to a better understanding of the relationship between anthropometric measures and disease progression. Although several studies have previously reported that taller and heavier young males with DMD are at higher risk of early loss of ambulation, this is the first systematic study exploring how changes in height and weight correlate with progression of motor performance over a relatively long follow‐up. These results confirm the importance of monitoring and managing weight for preserving motor function, and provide further information on changes in height that are a major concern in young males and young adults treated with glucocorticoids.

Length/height in DMD is known to be normal until the age of 2 to 3 years, showing a gradual slowdown during early years before and after glucocorticoid exposure.^4^ This suggests that more studies are needed to explore the interplay between bone and muscle biology. The impact of height on function is particularly relevant; a new generation of steroids (including vamorolone) are likely to have less impact on bone health and pubertal growth, reducing emotional distress associated with short stature and delayed puberty in adolescents and young adults with DMD.

The results of the study by Schiava et al. also included details on the impact of different steroid regimens. This will provide guidance for the management of glucocorticoids in future comparative studies, exploring the impact of these new drugs and the possibility of personalized approaches.

## Data Availability

Not required.
